# Early-Stage Rectal Mixed Neuroendocrine-Non-neuroendocrine Neoplasm Treated With Local Excision in a Frail Elderly Patient: A Multidisciplinary Management Dilemma

**DOI:** 10.7759/cureus.108141

**Published:** 2026-05-02

**Authors:** Christopher M Ahmad, Rae-Anne Kastle, Talyn Smith, Harsha Kalagana, Kerry Williams-Wuch

**Affiliations:** 1 Internal Medicine, Kansas City University, Joplin, USA; 2 General Surgery, Kansas City University, Joplin, USA; 3 Family Medicine, Kansas City University, Joplin, USA; 4 Hematology and Oncology, Mercy Hospital, Joplin, USA

**Keywords:** early-stage rectal cancer, frailty, lymphovascular invasion, mixed neuroendocrine-non-neuroendocrine neoplasm, multidisciplinary tumor board, rectal minen, surveillance management, transanal endoscopic microsurgery

## Abstract

Mixed neuroendocrine-non-neuroendocrine neoplasms (MiNENs) of the rectum are rare tumors that typically demonstrate aggressive behavior, largely driven by high-grade neuroendocrine carcinoma components. Evidence guiding management is limited, particularly for early-stage lesions and for patients unable to tolerate standard oncologic surgery. We report the case of an elderly man with extensive comorbidities who was found to have a 1.3 cm rectal MiNEN following transanal endoscopic microsurgery (TEMS) performed for rectal bleeding. Histopathology revealed a moderately differentiated adenocarcinoma component and a well-differentiated grade 1 neuroendocrine tumor (NET) component, with submucosal invasion greater than 2 mm, negative margins, low tumor budding, small-vessel lymphovascular invasion (LVI), and intact mismatch repair protein expression. No lymph nodes were sampled. Given the patient’s extensive comorbidities, progressive neurologic disease (including Parkinsonism and dementia), and reduced functional independence, he was considered a high-risk candidate for radical oncologic resection. The patient’s neurologic impairment and overall functional status were the primary factors limiting surgical escalation, rather than isolated cardiometabolic comorbidities alone. A surveillance-based approach with external expert pathology review was recommended. This case highlights the complexity of managing early-stage rectal MiNENs in medically vulnerable patients and underscores the importance of individualized, multidisciplinary decision-making when guideline-directed therapy is not feasible.

## Introduction

Mixed neuroendocrine-non-neuroendocrine neoplasms (MiNENs) are uncommon gastrointestinal malignancies defined by the presence of both neuroendocrine and non-neuroendocrine components within the same tumor, each typically comprising a substantial proportion of the lesion [1]. In the colorectum, MiNENs account for less than 1% of colorectal malignancies, and population-level incidence estimates remain poorly defined due to their rarity and evolving diagnostic criteria [2]. Despite increasing recognition, precise epidemiologic rates per population remain inconsistently reported across regions, further contributing to variability in clinical understanding and management.

The aggressive reputation of colorectal MiNENs largely reflects their frequent association with high-grade neuroendocrine carcinoma components, which are present in the majority of reported cases and drive early metastatic spread and poor prognosis [3]. Rectal MiNENs are particularly rare, and early-stage lesions containing a well-differentiated neuroendocrine tumor (NET) component are infrequently described [4-6]. As a result, standardized management strategies, especially for early-stage disease, remain poorly defined.

Clinical decision-making becomes especially challenging when oncologic principles favor aggressive intervention, but patient-related factors limit feasibility. This report describes an early-stage rectal MiNEN incidentally identified after local excision in a frail elderly patient and illustrates how multidisciplinary evaluation can guide individualized care when standard surgical management is not possible [7]. In this context, local excision techniques such as transanal endoscopic microsurgery (TEMS) may be considered in select patients, although their role in MiNEN remains poorly defined.

## Case presentation

**Figure 1:**
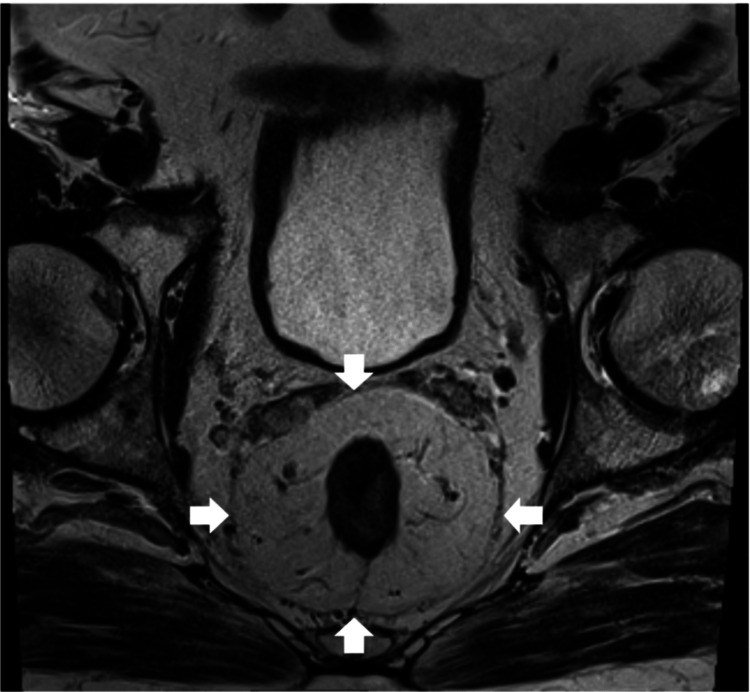
Axial pelvic contrast-enhanced computed tomography demonstrating a circumferential rectal lesion (arrows) without evidence of distant invasion.

An elderly man with a medical history significant for Parkinsonism, dementia, type 2 diabetes mellitus, hypertension, hyperlipidemia, benign prostatic hyperplasia, obstructive sleep apnea, chronic pain, major depressive disorder, and rapid eye movement (REM) sleep behavior disorder presented with rectal bleeding. Evaluation with a contrast computed tomography (CT) scan identified a rectal mass amenable to local excision (Figure 1).

**Figure 2:**
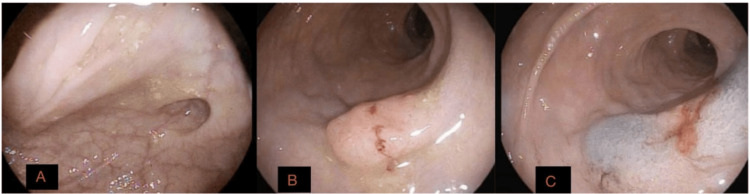
Colonoscopic identification and localization of rectal lesion. (A) Colonoscopic view demonstrating a sessile, approximately 2 cm lesion in the mid-rectum without active bleeding. (B) Closer endoscopic view of the rectal lesion demonstrating mucosal disruption following biopsy sampling. (C) An India ink tattoo placed adjacent to the lesion to facilitate localization prior to transanal endoscopic microsurgical excision.

Colonoscopy performed in July 2025 revealed a sessile approximately 2 cm mass in the mid-rectum without active bleeding (Figure 2), which measured 1.3 cm on final pathologic examination. The remainder of the colon appeared normal, except for mild sigmoid diverticulosis. Submucosal lift using Eleview was attempted but unsuccessful, and biopsies were obtained with cold forceps. The lesion was subsequently tattooed with India ink to facilitate localization for surgical excision.

**Figure 3:**
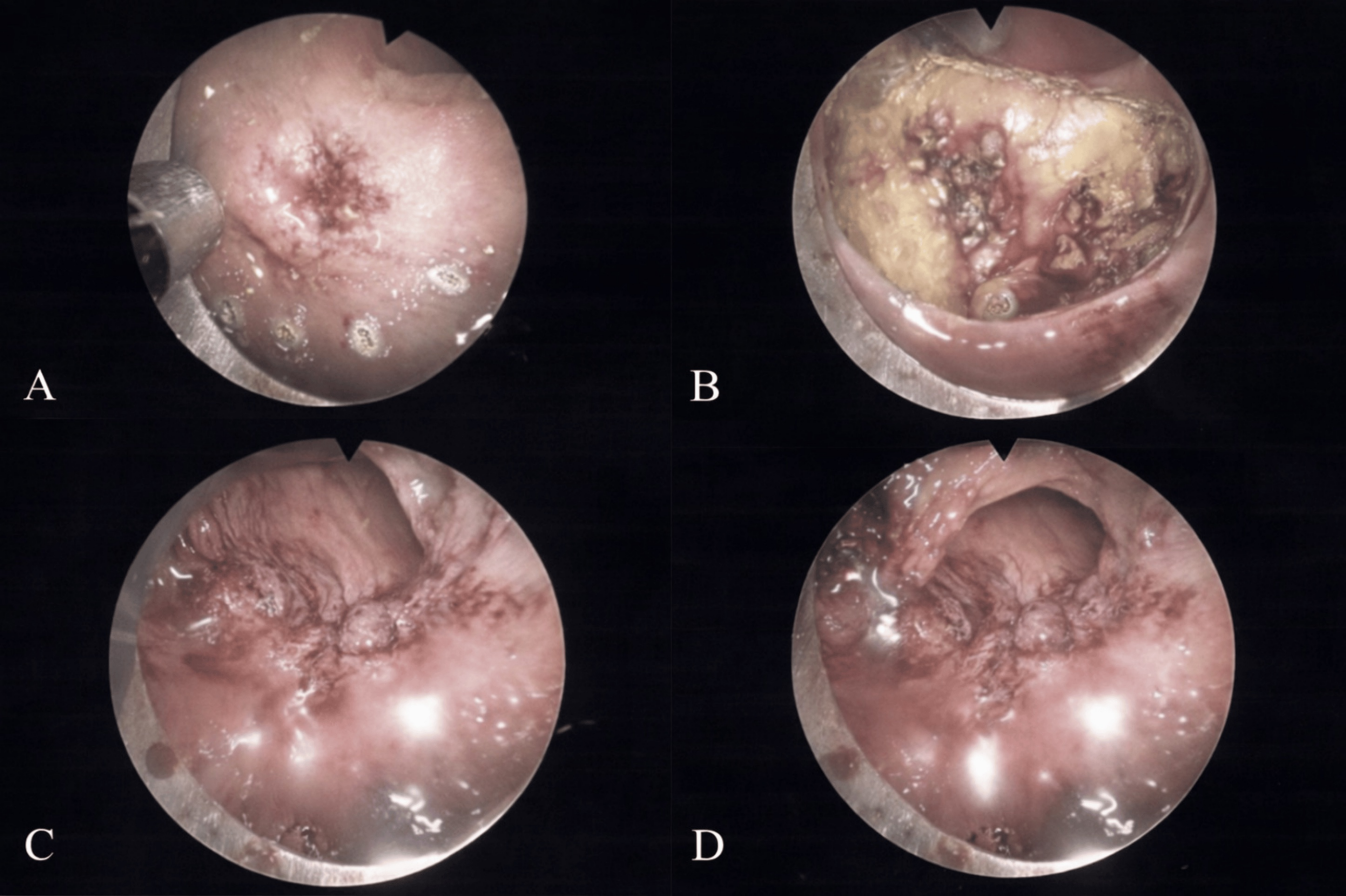
Transanal endoscopic microsurgical excision of a 1.3 cm MiNEN located rectally. (A) Initial endoscopic visualization of the rectal lesion. (B) Intraoperative view during transanal endoscopic microsurgical dissection. (C, D) Post-excision operative field demonstrating complete local resection. MiNEN: mixed neuroendocrine-non-neuroendocrine neoplasm.

Given the patient’s extensive comorbidities, progressive neurologic disease, and reduced functional independence, TEM was selected in lieu of radical oncologic resection. Histopathologic analysis demonstrated a 1.3 cm MiNEN. The non-neuroendocrine component consisted of invasive moderately differentiated adenocarcinoma (grade 2), while the neuroendocrine component was classified as a well-differentiated, grade 1 NET. Each component was estimated to comprise at least 30% of the tumor, meeting diagnostic criteria for MiNEN. The lesion invaded the submucosa to a depth exceeding 2 mm without involvement of the muscularis propria (Figure 3).

All surgical margins were negative, including a 0.6 cm deep margin and a 0.8 cm lateral mucosal margin. These measurements support complete local excision; however, the presence of lymphovascular invasion (LVI) introduces residual concern for occult nodal disease despite negative margins. Small-vessel LVI was identified, while perineural invasion was absent. Tumor budding was low. No lymph nodes were included in the specimen. Immunohistochemical analysis demonstrated intact mismatch repair protein expression. Key histopathologic findings that informed oncologic risk assessment are summarized in Table 1.

**Table 1:** Histopathologic characteristics of the rectal mixed neuroendocrine-non-neuroendocrine neoplasm (MiNEN). Pathologic features identified on local excision that informed oncologic risk assessment and highlighted uncertainty regarding optimal management in early-stage rectal MiNEN. LVI and submucosal invasion >2 mm are recognized as adverse pathologic features associated with increased nodal metastatic risk in early rectal malignancies. NET: neuroendocrine tumor, MMR: mismatch repair.

Feature	Finding
Tumor type	Mixed neuroendocrine-non-neuroendocrine neoplasm (MiNEN)
Tumor size	Small, early-stage lesion (1.3 cm)
Components	>30% well-differentiated NET (G1) + moderately differentiated adenocarcinoma (G2) (non-neuroendocrine)
Depth of invasion	Submucosa, >2 mm (pT1b/pT2 equivalent)
Ki-67 proliferation index	Low-moderate
Muscularis propria	Not involved
Surgical margins	Negative (deep: 0.6 cm; lateral: 0.8 cm)
Lymphovascular invasion (LVI)	Present (small vessel)
Perineural invasion	Absent
Tumor budding	Low (0-4 buds/0.785 mm^2^)
Nodal status	cN0 (no nodes sampled)
MMR gene status	Intact (MLH1, MSH2, MSH6, PMS2)
Final pathology stage	T2 (based on depth + synoptic documentation)

**Figure 4:**
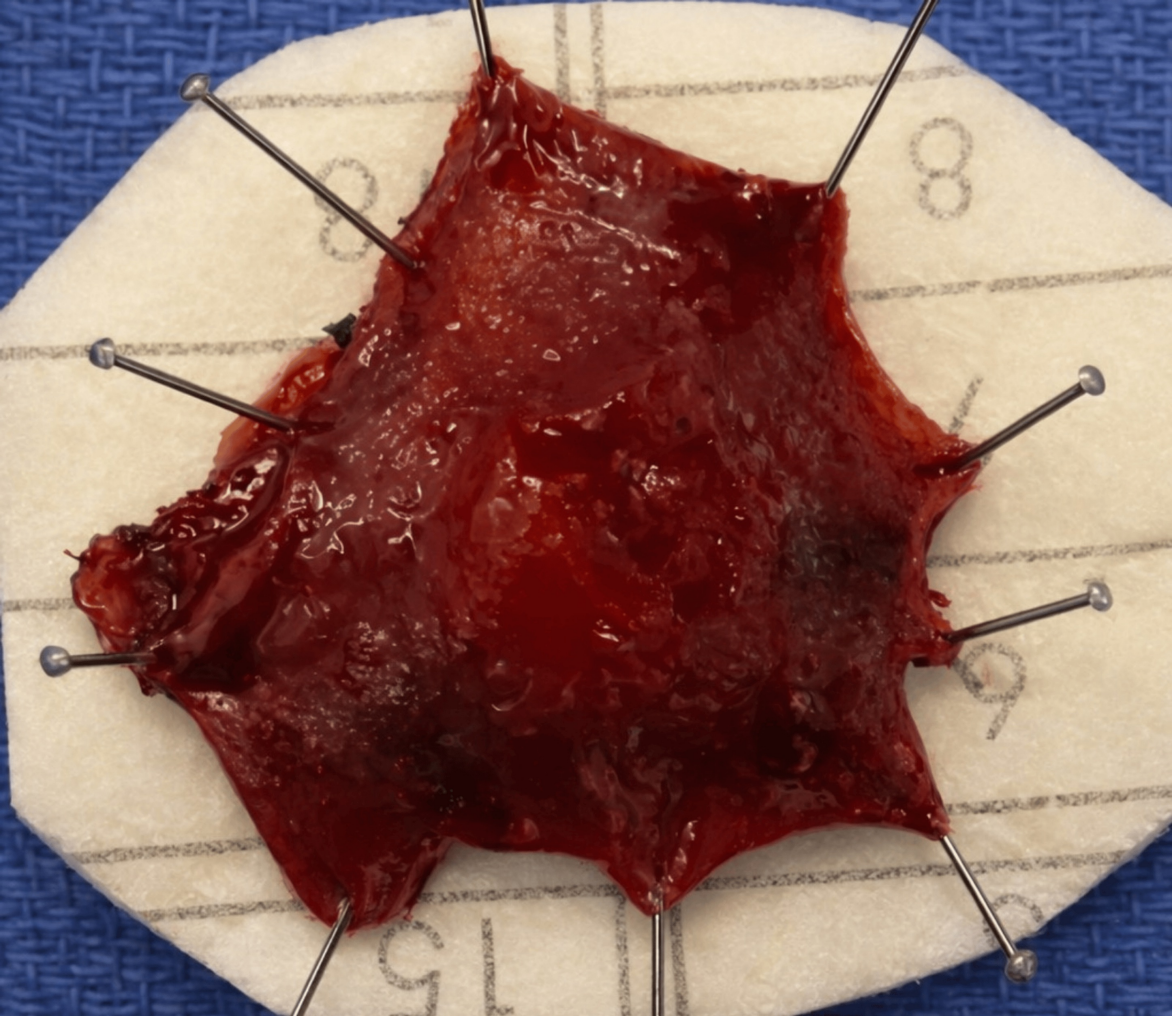
Gross specimen after transanal endoscopic microsurgical excision of rectal MiNEN. Oriented full-thickness rectal specimen pinned for margin assessment, measuring approximately 3 cm in maximal pinned dimension. Histopathology confirmed a 1.3 cm mixed neuroendocrine-non-neuroendocrine neoplasm (MiNEN) confined to the submucosa with negative margins. The apparent discrepancy between specimen size and tumor size reflects the inclusion of the surrounding rectal wall in the excision specimen.

**Figure 5:**
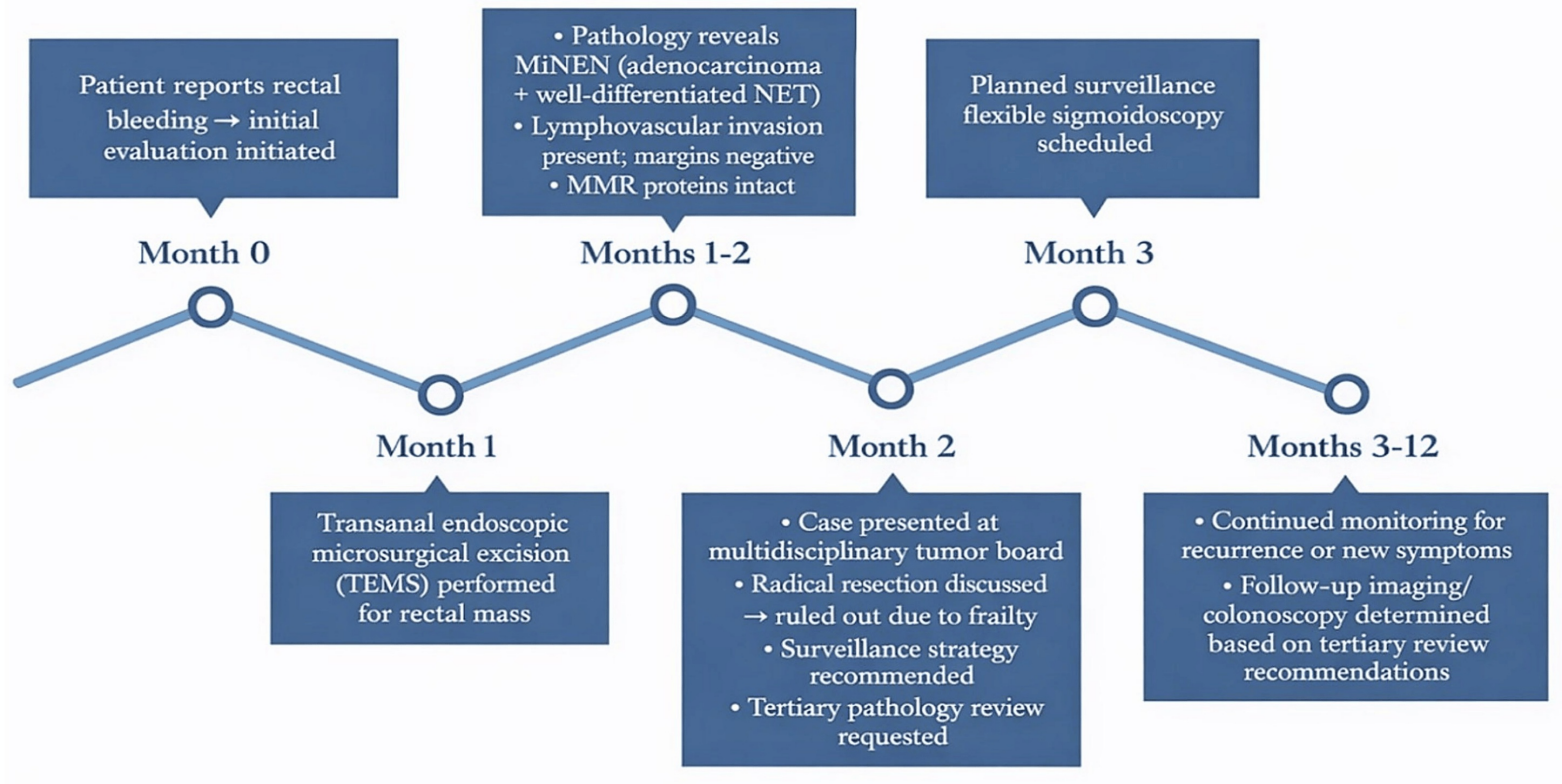
Diagnostic and management timeline. Diagnostic and management timeline from initial presentation to tumor board decision-making for an elderly man with early-stage rectal MiNEN. The figure illustrates the sequence of events leading to diagnosis, pathology review, and the final surveillance-based plan. MiNEN: mixed neuroendocrine-non-neuroendocrine neoplasm, NET: neuroendocrine tumor, MMR: mismatch repair. Image Credit: This is the authors' original work, created using BioRender (BioRender.com, Toronto, ON, Canada), with no artificial intelligence (AI) components.

The resected specimen was spread and oriented to facilitate assessment of the margins; the gross appearance is illustrated in Figure 4. Given the unusual pathology and the patient’s overall health status, the case was reviewed at a multidisciplinary tumor board including colorectal surgery, medical oncology, radiation oncology, gastroenterology, and pathology. While the absence of muscularis propria invasion and negative margins supported the adequacy of local excision, the MiNEN diagnosis and presence of LVI raised concern for occult metastatic risk. Radical resection with nodal sampling was discussed but deemed unsafe due to high operative risk. The consensus recommendation was close endoscopic surveillance and referral for external expert pathology review to confirm tumor classification and guide further management. At the time of decision-making, the patient required significant assistance with activities of daily living, further supporting a conservative management approach. The structured multidisciplinary decision-making framework is outlined in Table 2, and the diagnostic timeline is illustrated in Figure 5.

**Table 2:** Multidisciplinary decision-making framework for individualized care. Clinical decision-making framework used by the tumor board, balancing oncologic standards with real-world patient factors. The presence of LVI and submucosal invasion >2 mm increased theoretical concern for occult nodal disease despite negative margins. TEMS: transanal endoscopic microsurgery, LVI: lymphovascular invasion, NET: neuroendocrine tumor.

Domain	Considerations	Influence on management
Tumor biology	Early stage; presence of a well-differentiated neuroendocrine component	Supported local control via TEMS
Patient factors	Frailty, advanced age, comorbidities, mental status/functionality	Contraindicated radical proctectomy
Oncologic risk	LVI present; >2 mm submucosal invasion	Increased theoretical risk of nodal disease
Pathology review	Diagnostic complexity; rarity of G1 NET component	Triggered referral for tertiary expert review
Final consensus	Balance of safety vs. oncologic radicality	Recommendation: close surveillance

## Discussion

**Figure 6:**
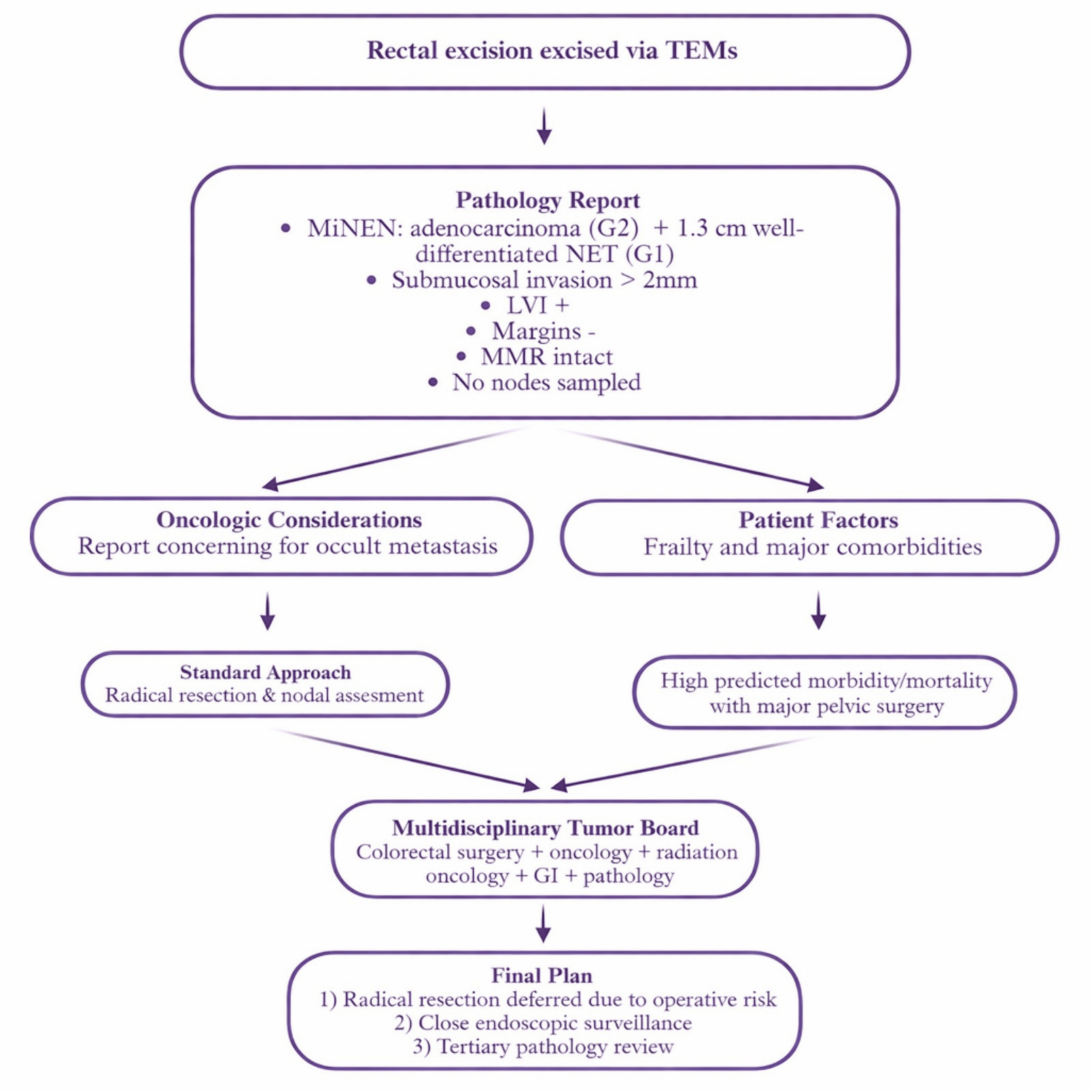
Management algorithm for early-stage rectal MiNEN in a frail patient. Decision-making algorithm showing how tumor biology, patient frailty, and multidisciplinary input shaped the management strategy for this rare early-stage rectal MiNEN. MiNEN: mixed neuroendocrine-non-neuroendocrine neoplasm, NET: neuroendocrine tumor, MMR: mismatch repair, TEMS: transanal endoscopic microsurgery, LVI: lymphovascular invasion, GI: gastrointestinal. Image Credit: This is the authors' original work, created using BioRender (BioRender.com, Toronto, ON, Canada), with no artificial intelligence (AI) components.

Rectal MiNENs are rare, and the majority of reported cases involve high-grade neuroendocrine carcinoma components that dominate prognosis and dictate aggressive treatment [8,9]. In contrast, this case involved a well-differentiated NET component alongside moderately differentiated adenocarcinoma, a combination that introduces prognostic and therapeutic uncertainty. A conceptual management algorithm derived from this case is shown in Figure 6.

The role of local excision in MiNENs remains poorly established. While local excision may be curative for select early-stage rectal adenocarcinomas or well-differentiated NETs, its applicability to MiNENs remains unclear [8,9]. Even small MiNENs may carry a disproportionate risk of lymphatic spread due to their mixed histology, with reported cases demonstrating metastatic potential even in lesions measuring less than 2 cm [8]. In this case, submucosal invasion greater than 2 mm and LVI increased concern for nodal disease, yet no lymph nodes were sampled. Under standard oncologic management, these features would typically favor radical resection with lymph node evaluation rather than local excision alone. In early rectal adenocarcinoma, LVI and submucosal invasion beyond 1-2 mm are established predictors of nodal metastasis, raising concern that a similar risk may extend to MiNENs despite limited direct evidence.

Patient-specific factors were central to management. Frailty, neurologic disease, and multimorbidity are well-recognized predictors of postoperative morbidity and mortality following major pelvic surgery [10,11]. In such patients, strict adherence to guideline-driven escalation may result in disproportionate harm. Multidisciplinary tumor board review allowed for balanced consideration of tumor biology, surgical risk, and patient-centered outcomes.

External expert pathology review was recommended, given the diagnostic complexity and known interobserver variability in MiNEN classification [9]. Accurate classification has important implications for prognosis, surveillance, and potential adjuvant strategies.

This case underscores the importance of documenting real-world management decisions in rare tumors, particularly when ideal oncologic pathways are constrained by patient-specific factors. In addition to its diagnostic complexity, the case highlights the persistent tension between theoretical oncologic completeness and practical surgical feasibility. While radical resection with formal nodal dissection may provide more definitive staging and potential disease control, such intervention carries substantial physiologic demand and recovery burden. In patients with limited functional reserve, frailty, or significant comorbidity, the incremental oncologic benefit of escalation must be carefully weighed against the risks of postoperative complications, prolonged hospitalization, and potential loss of independence.

Accordingly, decision-making in this context extends beyond tumor characteristics alone. It requires thoughtful integration of biologic risk, operative stress, anticipated quality-of-life impact, and the patient’s broader health trajectory. In rare malignancies where standardized pathways are limited, management often reflects calibrated clinical judgment rather than strict algorithmic adherence. This individualized approach, though sometimes less definitive from a staging standpoint, may ultimately represent the most appropriate balance between oncologic intent and patient-centered care.

Standard management for rectal malignancies with high-risk features typically includes radical resection with lymph node sampling, which allows accurate staging and may reduce recurrence risk. However, reported outcomes for MiNENs remain heterogeneous, and robust survival data specific to early-stage disease are lacking. As such, management strategies are often extrapolated from adenocarcinoma and NET paradigms, introducing inherent limitations and potential selection bias. In addition, alternative strategies, including adjuvant therapy or intensified surveillance, remain poorly defined in this context, particularly for patients who are not candidates for radical surgery.

Limitations

This report is limited by its single-case design and lack of lymph node sampling, which prevents definitive pathologic staging and leaves the possibility of occult nodal disease unresolved. Although margins were negative and the muscularis propria was not involved, the presence of LVI and submucosal invasion greater than 2 mm introduces uncertainty regarding the true metastatic risk. Management decisions were heavily influenced by the patient’s neurologic impairment, reduced functional independence, and overall medical complexity, limiting generalizability to medically fit individuals. The surveillance-based approach should therefore be interpreted as context-dependent rather than broadly applicable. Additionally, long-term follow-up data are not yet available, precluding assessment of recurrence risk or oncologic durability.

## Conclusions

Early-stage rectal MiNENs with well-differentiated neuroendocrine components are rarely encountered, and their clinical behavior and optimal treatment approach remain uncertain. In this medically frail elderly patient, management with local excision followed by multidisciplinary review and surveillance represented a context-specific compromise between oncologic risk and operative feasibility, given limited guideline support. This approach should be interpreted as individualized to this patient’s clinical condition rather than broadly generalizable. Structured surveillance with clearly defined intervals is essential in such cases, although optimal protocols remain undefined.

This case highlights the diagnostic ambiguity that often accompanies MiNENs and underscores the difficulty of applying treatment paradigms derived from more common colorectal or NETs. Continued reporting of early-stage MiNEN cases, particularly in patients with significant comorbidity, is necessary to improve diagnostic clarity, refine prognostic understanding, and guide management strategies for medically vulnerable populations. The development of multicenter registries for early-stage MiNENs may be critical to advancing evidence-based care for this rare and heterogeneous disease.
